# Supplementary material for: DNA vaccine candidate encoding SARS-CoV-2 spike proteins elicited potent humoral and Th1 cell-mediated immune responses in mice
Source: PLoS One. 2021 Mar 22;16(3):e0248007. doi: 10.1371/journal.pone.0248007 (PMC7984610; doi:10.1371/journal.pone.0248007)
Supplement: S1 Data — (DOCX) [file pone.0248007.s001.docx]

**S1 Data**

1. **SARS-CoV-2 Spike (S) nucleotide sequence**

ATGTTCGTGTTTCTGGTCCTGCTGCCCCTGGTCTCTTCTCAGTGCGTGAACCTGACAACTCGGACTCAGCTGCCTCCTGCCTATACTAATTCCTTCACCCGGGGCGTGTACTATCCTGACAAGGTGTTTAGAAGCTCCGTGCTGCACTCCACACAGGATCTGTTTCTGCCATTCTTTTCTAACGTGACCTGGTTCCACGCCATCCACGTGAGCGGCACCAATGGCACAAAGCGGTTCGACAATCCCGTGCTGCCTTTTAACGATGGCGTGTACTTCGCCTCCACCGAGAAGTCTAACATCATCAGAGGCTGGATCTTTGGCACCACACTGGACAGCAAGACACAGTCCCTGCTGATCGTGAACAATGCCACCAACGTGGTCATCAAGGTGTGCGAGTTCCAGTTTTGTAATGATCCCTTCCTGGGCGTGTACTATCACAAGAACAATAAGTCTTGGATGGAGAGCGAGTTTAGAGTGTATTCTAGCGCCAACAATTGCACATTTGAGTACGTGTCCCAGCCTTTCCTGATGGACCTGGAGGGCAAGCAGGGCAATTTCAAGAACCTGAGGGAGTTCGTGTTTAAGAATATCGATGGCTACTTCAAAATCTACTCCAAGCACACCCCCATCAACCTGGTGCGCGACCTGCCTCAGGGCTTCTCTGCCCTGGAGCCCCTGGTGGATCTGCCTATCGGCATCAACATCACCCGGTTTCAGACACTGCTGGCCCTGCACAGAAGCTACCTGACACCCGGCGACTCCTCTAGCGGATGGACCGCAGGAGCTGCCGCCTACTATGTGGGCTATCTGCAGCCTAGGACCTTCCTGCTGAAGTACAACGAGAATGGCACCATCACAGACGCAGTGGATTGCGCCCTGGACCCCCTGTCTGAGACAAAGTGTACACTGAAGAGCTTTACCGTGGAGAAGGGCATCTATCAGACAAGCAATTTCAGGGTGCAGCCAACCGAGTCCATCGTGCGCTTTCCTAATATCACAAACCTGTGCCCATTTGGCGAGGTGTTCAACGCAACCAGGTTCGCCAGCGTGTACGCATGGAATAGGAAGCGCATCTCCAACTGCGTGGCCGACTATTCTGTGCTGTACAACTCCGCCTCTTTCAGCACCTTTAAGTGCTATGGCGTGAGCCCCACAAAGCTGAATGACCTGTGCTTTACCAACGTGTACGCCGATTCCTTCGTGATCAGGGGCGACGAGGTGCGCCAGATCGCACCTGGACAGACAGGCAAGATCGCCGACTACAATTATAAGCTGCCAGACGATTTCACCGGCTGCGTGATCGCCTGGAACTCTAACAATCTGGATAGCAAAGTGGGCGGCAACTACAATTATCTGTACCGGCTGTTTAGAAAGTCTAATCTGAAGCCCTTCGAGAGGGACATCTCCACAGAAATCTACCAGGCCGGCTCTACCCCTTGCAATGGCGTGGAGGGCTTTAACTGTTATTTCCCACTGCAGAGCTACGGCTTCCAGCCCACAAACGGCGTGGGCTATCAGCCTTACCGCGTGGTGGTGCTGTCTTTTGAGCTGCTGCACGCACCAGCAACAGTGTGCGGACCCAAGAAGAGCACCAATCTGGTGAAGAACAAGTGCGTGAACTTCAACTTCAACGGCCTGACCGGCACAGGCGTGCTGACCGAGTCCAACAAGAAGTTCCTGCCATTTCAGCAGTTCGGCAGGGACATCGCAGATACCACAGACGCCGTGCGCGACCCACAGACCCTGGAGATCCTGGACATCACACCCTGCTCCTTCGGCGGCGTGTCTGTGATCACACCCGGCACCAATACAAGCAACCAGGTGGCCGTGCTGTATCAGGACGTGAATTGTACCGAGGTGCCTGTGGCCATCCACGCCGATCAGCTGACCCCAACATGGCGGGTGTACAGCACCGGCTCCAACGTGTTCCAGACAAGAGCCGGATGCCTGATCGGAGCAGAGCACGTGAACAATAGCTATGAGTGCGACATCCCAATCGGCGCCGGCATCTGTGCCTCCTACCAGACCCAGACAAACTCCCCAAGGAGAGCACGGTCTGTGGCCTCCCAGTCTATCATCGCCTATACCATGAGCCTGGGCGCCGAGAACAGCGTGGCCTACTCTAACAATAGCATCGCCATCCCAACCAACTTCACAATCTCCGTGACCACAGAGATCCTGCCCGTGAGCATGACCAAGACATCCGTGGACTGCACAATGTATATCTGTGGCGATTCCACCGAGTGCTCTAACCTGCTGCTGCAGTACGGCTCTTTTTGTACCCAGCTGAATAGAGCCCTGACAGGCATCGCCGTGGAGCAGGATAAGAACACACAGGAGGTGTTCGCCCAGGTGAAGCAAATCTACAAGACCCCCCCTATCAAGGACTTTGGCGGCTTCAATTTTTCCCAGATCCTGCCTGATCCATCCAAGCCTTCTAAGCGGAGCTTTATCGAGGACCTGCTGTTCAACAAGGTGACCCTGGCCGATGCCGGCTTCATCAAGCAGTATGGCGATTGCCTGGGCGACATCGCAGCCCGGGACCTGATCTGCGCCCAGAAGTTTAATGGCCTGACCGTGCTGCCACCCCTGCTGACAGATGAGATGATCGCCCAGTACACATCTGCCCTGCTGGCCGGCACCATCACAAGCGGATGGACCTTCGGCGCAGGAGCCGCCCTGCAGATCCCCTTTGCCATGCAGATGGCCTATAGGTTCAACGGCATCGGCGTGACCCAGAATGTGCTGTACGAGAACCAGAAGCTGATCGCCAATCAGTTTAACTCCGCCATCGGCAAGATCCAGGACTCTCTGTCCTCTACAGCCAGCGCCCTGGGCAAGCTGCAGGATGTGGTGAATCAGAACGCCCAGGCCCTGAATACCCTGGTGAAGCAGCTGAGCTCCAACTTCGGCGCCATCTCTAGCGTGCTGAATGACATCCTGAGCCGGCTGGACAAGGTGGAGGCAGAGGTGCAGATCGACCGGCTGATCACAGGCAGACTGCAGTCCCTGCAGACCTATGTGACACAGCAGCTGATCAGGGCAGCAGAGATCAGGGCCTCTGCCAATCTGGCCGCCACCAAGATGAGCGAGTGCGTGCTGGGCCAGTCCAAGAGAGTGGACTTTTGTGGCAAGGGCTATCACCTGATGAGCTTCCCTCAGTCCGCCCCACACGGCGTGGTGTTTCTGCACGTGACCTACGTGCCCGCCCAGGAGAAGAACTTCACCACAGCCCCTGCCATCTGCCACGATGGCAAGGCCCACTTTCCAAGGGAGGGCGTGTTCGTGAGCAACGGCACCCACTGGTTTGTGACACAGCGCAATTTCTACGAGCCCCAGATCATCACCACAGACAATACCTTCGTGAGCGGCAACTGTGACGTGGTCATCGGCATCGTGAACAATACCGTGTATGATCCACTGCAGCCCGAGCTGGACTCTTTTAAGGAGGAGCTGGATAAGTACTTCAAGAATCACACCAGCCCTGACGTGGATCTGGGCGACATCTCTGGCATCAATGCCAGCGTGGTGAACATCCAGAAGGAGATCGACCGCCTGAACGAGGTGGCCAAGAATCTGAACGAGAGCCTGATCGATCTGCAGGAGCTGGGCAAGTATGAGCAGTACATCAAGTGGCCCTGGTACATCTGGCTGGGCTTCATCGCCGGCCTGATCGCTATCGTGATGGTGACTATTATGCTGTGCTGTATGACCTCCTGCTGTTCTTGTCTGAAGTAA

1. **SARS-CoV-2 S1 nucleotide sequence**

ATGTTCGTGTTTCTGGTCCTGCTGCCCCTGGTCTCTTCTCAGTGCGTGAACCTGACAACTCGGACTCAGCTGCCTCCTGCCTATACTAATTCCTTCACCCGGGGCGTGTACTATCCTGACAAGGTGTTTAGAAGCTCCGTGCTGCACTCCACACAGGATCTGTTTCTGCCATTCTTTTCTAACGTGACCTGGTTCCACGCCATCCACGTGAGCGGCACCAATGGCACAAAGCGGTTCGACAATCCCGTGCTGCCTTTTAACGATGGCGTGTACTTCGCCTCCACCGAGAAGTCTAACATCATCAGAGGCTGGATCTTTGGCACCACACTGGACAGCAAGACACAGTCCCTGCTGATCGTGAACAATGCCACCAACGTGGTCATCAAGGTGTGCGAGTTCCAGTTTTGTAATGATCCCTTCCTGGGCGTGTACTATCACAAGAACAATAAGTCTTGGATGGAGAGCGAGTTTAGAGTGTATTCTAGCGCCAACAATTGCACATTTGAGTACGTGTCCCAGCCTTTCCTGATGGACCTGGAGGGCAAGCAGGGCAATTTCAAGAACCTGAGGGAGTTCGTGTTTAAGAATATCGATGGCTACTTCAAAATCTACTCCAAGCACACCCCCATCAACCTGGTGCGCGACCTGCCTCAGGGCTTCTCTGCCCTGGAGCCCCTGGTGGATCTGCCTATCGGCATCAACATCACCCGGTTTCAGACACTGCTGGCCCTGCACAGAAGCTACCTGACACCCGGCGACTCCTCTAGCGGATGGACCGCAGGAGCTGCCGCCTACTATGTGGGCTATCTGCAGCCTAGGACCTTCCTGCTGAAGTACAACGAGAATGGCACCATCACAGACGCAGTGGATTGCGCCCTGGACCCCCTGTCTGAGACAAAGTGTACACTGAAGAGCTTTACCGTGGAGAAGGGCATCTATCAGACAAGCAATTTCAGGGTGCAGCCAACCGAGTCCATCGTGCGCTTTCCTAATATCACAAACCTGTGCCCATTTGGCGAGGTGTTCAACGCAACCAGGTTCGCCAGCGTGTACGCATGGAATAGGAAGCGCATCTCCAACTGCGTGGCCGACTATTCTGTGCTGTACAACTCCGCCTCTTTCAGCACCTTTAAGTGCTATGGCGTGAGCCCCACAAAGCTGAATGACCTGTGCTTTACCAACGTGTACGCCGATTCCTTCGTGATCAGGGGCGACGAGGTGCGCCAGATCGCACCTGGACAGACAGGCAAGATCGCCGACTACAATTATAAGCTGCCAGACGATTTCACCGGCTGCGTGATCGCCTGGAACTCTAACAATCTGGATAGCAAAGTGGGCGGCAACTACAATTATCTGTACCGGCTGTTTAGAAAGTCTAATCTGAAGCCCTTCGAGAGGGACATCTCCACAGAAATCTACCAGGCCGGCTCTACCCCTTGCAATGGCGTGGAGGGCTTTAACTGTTATTTCCCACTGCAGAGCTACGGCTTCCAGCCCACAAACGGCGTGGGCTATCAGCCTTACCGCGTGGTGGTGCTGTCTTTTGAGCTGCTGCACGCACCAGCAACAGTGTGCGGACCCAAGAAGAGCACCAATCTGGTGAAGAACAAGTGCGTGAACTTCAACTTCAACGGCCTGACCGGCACAGGCGTGCTGACCGAGTCCAACAAGAAGTTCCTGCCATTTCAGCAGTTCGGCAGGGACATCGCAGATACCACAGACGCCGTGCGCGACCCACAGACCCTGGAGATCCTGGACATCACACCCTGCTCCTTCGGCGGCGTGTCTGTGATCACACCCGGCACCAATACAAGCAACCAGGTGGCCGTGCTGTATCAGGACGTGAATTGTACCGAGGTGCCTGTGGCCATCCACGCCGATCAGCTGACCCCAACATGGCGGGTGTACAGCACCGGCTCCAACGTGTTCCAGACAAGAGCCGGATGCCTGATCGGAGCAGAGCACGTGAACAATAGCTATGAGTGCGACATCCCAATCGGCGCCGGCATCTGTGCCTCCTACCAGACCCAGACAAACTCCCCAAGGAGAGCACGGTAA

1. **SARS-CoV-2 S2 nucleotide sequence**

ATGTTCGTGTTTCTGGTCCTGCTGCCCCTGGTCTCTTCTCAGTGCTCTGTGGCCTCCCAGTCTATCATCGCCTATACCATGAGCCTGGGCGCCGAGAACAGCGTGGCCTACTCTAACAATAGCATCGCCATCCCAACCAACTTCACAATCTCCGTGACCACAGAGATCCTGCCCGTGAGCATGACCAAGACATCCGTGGACTGCACAATGTATATCTGTGGCGATTCCACCGAGTGCTCTAACCTGCTGCTGCAGTACGGCTCTTTTTGTACCCAGCTGAATAGAGCCCTGACAGGCATCGCCGTGGAGCAGGATAAGAACACACAGGAGGTGTTCGCCCAGGTGAAGCAAATCTACAAGACCCCCCCTATCAAGGACTTTGGCGGCTTCAATTTTTCCCAGATCCTGCCTGATCCATCCAAGCCTTCTAAGCGGAGCTTTATCGAGGACCTGCTGTTCAACAAGGTGACCCTGGCCGATGCCGGCTTCATCAAGCAGTATGGCGATTGCCTGGGCGACATCGCAGCCCGGGACCTGATCTGCGCCCAGAAGTTTAATGGCCTGACCGTGCTGCCACCCCTGCTGACAGATGAGATGATCGCCCAGTACACATCTGCCCTGCTGGCCGGCACCATCACAAGCGGATGGACCTTCGGCGCAGGAGCCGCCCTGCAGATCCCCTTTGCCATGCAGATGGCCTATAGGTTCAACGGCATCGGCGTGACCCAGAATGTGCTGTACGAGAACCAGAAGCTGATCGCCAATCAGTTTAACTCCGCCATCGGCAAGATCCAGGACTCTCTGTCCTCTACAGCCAGCGCCCTGGGCAAGCTGCAGGATGTGGTGAATCAGAACGCCCAGGCCCTGAATACCCTGGTGAAGCAGCTGAGCTCCAACTTCGGCGCCATCTCTAGCGTGCTGAATGACATCCTGAGCCGGCTGGACAAGGTGGAGGCAGAGGTGCAGATCGACCGGCTGATCACAGGCAGACTGCAGTCCCTGCAGACCTATGTGACACAGCAGCTGATCAGGGCAGCAGAGATCAGGGCCTCTGCCAATCTGGCCGCCACCAAGATGAGCGAGTGCGTGCTGGGCCAGTCCAAGAGAGTGGACTTTTGTGGCAAGGGCTATCACCTGATGAGCTTCCCTCAGTCCGCCCCACACGGCGTGGTGTTTCTGCACGTGACCTACGTGCCCGCCCAGGAGAAGAACTTCACCACAGCCCCTGCCATCTGCCACGATGGCAAGGCCCACTTTCCAAGGGAGGGCGTGTTCGTGAGCAACGGCACCCACTGGTTTGTGACACAGCGCAATTTCTACGAGCCCCAGATCATCACCACAGACAATACCTTCGTGAGCGGCAACTGTGACGTGGTCATCGGCATCGTGAACAATACCGTGTATGATCCACTGCAGCCCGAGCTGGACTCTTTTAAGGAGGAGCTGGATAAGTACTTCAAGAATCACACCAGCCCTGACGTGGATCTGGGCGACATCTCTGGCATCAATGCCAGCGTGGTGAACATCCAGAAGGAGATCGACCGCCTGAACGAGGTGGCCAAGAATCTGAACGAGAGCCTGATCGATCTGCAGGAGCTGGGCAAGTATGAGCAGTACATCAAGTGGCCCTGGTACATCTGGCTGGGCTTCATCGCCGGCCTGATCGCTATCGTGATGGTGACTATTATGCTGTGCTGTATGACCTCCTGCTGTTCTTGTCTGAAGTAA
