# Supplementary material for: DNA vaccine candidate encoding SARS-CoV-2 spike proteins elicited potent humoral and Th1 cell-mediated immune responses in mice
Source: PLoS One. 2021 Mar 22;16(3):e0248007. doi: 10.1371/journal.pone.0248007 (PMC7984610; doi:10.1371/journal.pone.0248007)
Supplement: Attachment — Submitted filename: Response to reviewers PONE-D-20-38402.docx [file pone.0248007.s002.docx]

Re- PONE-D-20-38402

Dear Editor,

Thank you very much for the useful comments from editor and reviewers. On behalf of the authors, I’d like to respond as following:

***Editor comments:***

1. Please, consider changing the title as suggested by reviewer #2;

**Response**: the title was revised to **“DNA vaccine candidate encoding SARS-CoV-2 spike proteins elicited potent humoral and Th1 cell-mediated immune responses in mice”**

2) The authors should provide more methodological details in order to allow others to repeat and confirm the results;

**Response**: See the responses on the methodological details to both Reviewer#1 and Reviewer#2 below

3) Please, answer to all the comments raised by the both reviewers.
**Response**: See the response to both Reviewer#1 and Reviewer#2 below

***Reviewer#1***

- Line 122 - Plasmids transfection and in vitro protein expression analysis - Protocols must be better described. Please, inform with more details the quantities of reagents were used in this assays. How much cells were transfected??? How much plasmid, lipofectamine and IgG-FITC were used???

**Response:** The details for plasmid transfection and *in vitro* protein expression was described*.*

“At 24 hr before transfection, 1x10^6^ cells of HEK293T were seeded in 6-well plate (Thermo Fisher Scientific, MA, USA). The cells with approximately 80-90% confluency were separately transfected with individual recombinant plasmid constructs (pCMVkan-S, -S1 and -S2) using lipofectamine 3000™ (Invitrogen, Carlsbad, CA, USA) according to the manufacture’s protocol. Briefly, 2.5 µg of plasmid and 3.75 µL lipofectamine 3000™ were separately diluted in 125 µL and 250 µL serum-free Opti-MEM™ medium (Gibco), respectively. P3000™, a transfection enhancer reagent provided in lipofectamine 3000™ kit, was also added into diluted plasmid tube to a final concentration of 2 µL/µg DNA. The diluted DNA was mixed with the diluted lipofectamine 3000™ at 1:1 ratio (v/v) and incubated for 15 min at room temperature. The plasmid-lipofectamine 3000™ complex was then added onto cells.” *(Line 136-146)*

- Line 131 Please include the average weight of the animals.

**Response:** Mouse weight (20-25 grams) was included *(Line 153).*

- Were the blood samples taken on the same day as the immunizations? Please clarify.
  **Response:** The detail for blood sample collection was clarified (*Line 160-161)****.***

“Blood samples were collected at the day before each immunization, and 2 and 4 weeks after the 3^rd^ dose designated as weeks 0, 2, 4, 6 and 8.”

- Line 140 - ELISA:
  Please, inform antigen concentrations in micrograms/well used. How much of sera was used, 100 microliters? Please, specify.
  Please inform quantity (titer) of HRP anti-mouse IgG was used.
  How much of TMB was used???

**Response: The detail for ELISA protocol was provided in the method including**

- antigen concentrations in micrograms/well used

“In each well, the mixture of S1 (110 ng) and S2 (85 ng) which correspond to approximately 1.5 picomolar of each protein was diluted in 100 µL of coating buffer” *(Line 177-178)*

- amount of sera

*“*25 µL*” (Line 182)*

- quantity (titer) of HRP anti-mouse IgG

*“*1:5000 dilutions of each horseradish peroxidase (HRP)-conjugated secondary Abs*” (Line 183)*

- Amount of TMB used	100 µL of tetramethylbenzidine (TMB) substrate *(Line 186)*
- Line 158 - RBD-ACE2 binding inhibition
  How much of diluted mice sera (what dilution???) and RBD-HRP were used???

**Response:** The detail for RBD-ACE2 binding inhibition protocol was provided in the method including

- dilution of sera

diluted mice sera (1:100) *(Line 195)*

- the use of RBD-HRP

RBD conjugated with HRP *(Line 195)*

- Volume of TMB used

100 µL of TMB *(Line 197)*

- Volume of stop solution

50 µL of stop solution *(Line 198)*

- Line 282 : Please, describe what NHP means (non human primates???)

**Response:** Yes, NHP is stand for non-human primates (Line 316)

***Reviewer #2:***

- Prompetchara et al describe the production and the use of plasmids containing full-length, just the S1, or S2 portions of the S1-glycoprotein of SARS-Cov2 in generating immune responses in mice. Similar studies have shown the possibility to generate immune responses in mice, guinea pig and Rhesus monkey. Recently, one of them has been adapted to be used in humans (https://doi.org/10.1016/j.eclinm.2020.100689), which could be additionally cited by the authors.

**Response:** The recent study in phase I clinical is cited

“The study in phase I clinical trial demonstrated that DNA vaccine using full-length wild type spike was safe and could induce both antibodies and T cells responses (59)” *(Ref 59, Line 388-390)*

- The results show improved responses with the use of full length spike, which should be better highlighted in the manuscript.
  **Response:** We highlighted the use of wild-type full length which it could provide high level of protective efficacy as seen in ChAdOx1 nCoV-19 *(in discussion Line 392-39*6). And based on our findings, in conclusion we also state that full length S (S1+S2) is better than the truncated S1 or S2 immunogens *(Line 408-401).*
- I would replace the title for DNA plasmid as there is not in vivo challenging in this work. Otherwise, use vaccine candidate.
  **Response**: the title was revised to **“**DNA vaccine **candidate** encoding SARS-CoV-2 spike proteins elicited potent humoral and Th1 cell-mediated immune responses in mice”
- Line 38: define GMT in the abstract

**Response:** Geometric mean titers added *(Line 49)*

- Line 40: stimulation
  **Response:** corrected to stimulation *(Line 52)*
- Plasmid nucleotide sequence details or deposit in public data bank should be provided. It is remarkable that many works have not disclosed this information, which I fell incorrect in scientific publications.
  **Response:** As mentioned in method section, we used the sequence from online database which is the wild-type sequence without further modification of amino acids. So, the sequence still the same with the uploaded database.
- The reference of pCMVkan should be cited in methods.
  **Response:** The information of pCMVkan and reference was included

*“*pCMVkan expression vector which contain the cytomegalovirus promoter, the bovine growth hormone polyadenylation site, and the kanamycin-resistant gene (24)*” (Ref 24, Line 125-127)*

- Details about transfections (amounts and purification of DNA plasmids) are important to be mentioned here.
  **Response:** The details for plasmid transfection and *in vitro* protein expression was described

“At 24 hr before transfection, 1x10^6^ cells of HEK293T were seeded in 6-well plate (Thermo Fisher Scientific, MA, USA). The cells with approximately 80-90% confluency were separately transfected with individual recombinant plasmid constructs (pCMVkan-S, -S1 and -S2) using lipofectamine 3000™ (Invitrogen, Carlsbad, CA, USA) according to the manufacture’s protocol. Briefly, 2.5 µg of plasmid and 3.75 µL lipofectamine 3000™ were separately diluted in 125 µL and 250 µL serum-free Opti-MEM™ medium (Gibco), respectively. P3000™, a transfection enhancer reagent provided in lipofectamine 3000™ kit, was also added into diluted plasmid tube to a final concentration of 2 µL/µg DNA. The diluted DNA was mixed with the diluted lipofectamine 3000™ at 1:1 ratio (v/v) and incubated for 15 min at room temperature. The plasmid-lipofectamine 3000™ complex was then added onto cells.” *(Line 136-146),*

Purification of DNA plasmids procedure was mentioned

“The recombinant plasmids were propagated in *E. coli,* DH5-alpha (Invitrogen, Carlsbad, CA, USA) and purified by Qiagen endotoxin-free giga plasmid kit (Hilden, Germany) following the manufacturer’s protocol.”(*Line 130-133)*

- In house preparation of S2 should be further explained.
  **Response:** The procedure of S2 production and purification is described

*“*The S2 protein was prepared by induction of the plasmid pET15b encoding S2 region fused with 6HIS at N terminal. In brief, transformed *E. coli* BL21(*DE3*) were grown at 37°C in LB (Luria-Bertani) medium supplemented with 100 µg/mL ampicillin. When the OD_600_ of the cell culture reached 0.6, isopropyl β-D-1-thiogalactopyranoside, IPTG (final concentration 0.1 mM) was added to induce protein expression and cells were grown at 25°C for a further 3 h. Cells were harvest by centrifugation at 8,000 g for 10 min at 4°C. For S2 purification, the cell pellet was resuspended in PBS supplemented with DNase (10 µg/mL) and RNase (10 µg/mL). The cells were lysed by sonication on ice for 15 min. The lysed cells were then centrifuged at 40,000 rpm for 1 h, only supernatant was collected. S2 protein was purified by affinity chromatography using HisTrap column (GE Healthcare, Sweden). The concentration of purified protein was measured by using Pierce™ BCA protein assay kit (Thermo Fisher Scientific, MA, USA).*”(Line 165-176)*

- Fig. 2: size bars are poorly visible and
  **Response:** The bar size was improved
- Fig. 3: It is not clear which assay was used for IgG1xIgG2, please explain.

**Response:** IgG1 and IgG2a were analyzed by ELISA using secondary antibodies which specific to IgG1 and IgG2a subclasses. The method is mention in Line 182-185.

**“**The plates were then washed and incubated with 1:5000 dilutions of each horseradish peroxidase (HRP)-conjugated secondary Abs including rabbit anti-mouse IgG (KPL, USA), rabbit anti-mouse IgG1 or rabbit anti-mouse IgG2a (both Abs were from BioLegend, USA) for additional 1 h at 37°C.**”**
