# Supplementary material for: DNA vaccine candidate encoding SARS-CoV-2 spike proteins elicited potent humoral and Th1 cell-mediated immune responses in mice
Source: PLoS One. 2021 Mar 22;16(3):e0248007. doi: 10.1371/journal.pone.0248007 (PMC7984610; doi:10.1371/journal.pone.0248007)
Supplement: Attachment — Submitted filename: Response to reviewers PONE-D-20-38402 R2.docx [file pone.0248007.s003.docx]

Re- PONE-D-20-38402

Dear Editor,

Thank you very much for the useful comments from editor and reviewers. On behalf of the authors, I’d like to respond as following:

***Reviewer comment:***

1. The authors should provide a DNA sequence of the construct used for expression.

Response: The nucleotide sequences (humanized codons) of Spike (S), S1 and S2 are attached as supplementary data 1 (line 128).
